# Clinical-radiological comparative evaluation of the BISAP and Computed tomography severity index scores as predictors of severity in acute pancreatitis

**DOI:** 10.12669/pjms.42.9.15523

**Published:** 2026-09

**Authors:** Nasreen Aman, Nosheen Noor, Muhammad Hamza, Hamna Khalid

**Affiliations:** 1 Nasreen Aman, FCPS Radiology Associate Professor, Radiology Department, Lady Reading Hospital, Peshawar, Pakistan; 2 Nosheen Noor, FCPS Radiology Assistant Professor, Radiology Department, Lady Reading Hospital, Peshawar, Pakistan; 3 Muhammad Hamza, FCPS 2 Resident Radiology Department, Lady Reading Hospital, Peshawar, Pakistan; 4 Hamna Khalid, MBBS 4th year Student. Wah Medical College, Punjab, Pakistan

**Keywords:** Acute pancreatitis, BISAP score, CT severity index, ROC Curve

## Abstract

**Objectives:**

To compare the clinical and radiological performance of the Bedside Index for Severity in Acute Pancreatitis (BISAP) and the Computed Tomography Severity Index (CTSI) in predicting disease severity and mortality in patients with acute pancreatitis, providing locally relevant evidence from a tertiary care setting in Pakistan.

**Methodology:**

This retrospective cross-sectional study was conducted at the Department of Radiology, Lady Reading Hospital–MTI, Peshawar, from January to December 2024. Data from 99 adult patients diagnosed with acute pancreatitis according to the Revised Atlanta Criteria (2012) were analyzed. BISAP scores were calculated using clinical and laboratory parameters recorded within 24 hours of admission, while CTSI was derived from contrast-enhanced CT findings. Disease severity was dichotomized into severe and non-severe categories for receiver operating characteristic (ROC) curve analysis. Diagnostic accuracy, optimal cut-off values, and predictive performance of BISAP and CTSI for severity and in-hospital mortality were assessed.

**Results:**

Out of 99 patients, severe acute pancreatitis was identified in 35.4% by CTSI and 20.2% by BISAP scores. ROC analysis demonstrated superior predictive performance of BISAP scores for severe acute pancreatitis with an area under the curve (AUC) of 0.667 (95% CI: 0.559–0.775; p = 0.006), compared to CTSI scores with an AUC of 0.571 (95% CI: 0.456–0.686; p = 0.239). For mortality prediction, BISAP scores showed better discrimination (AUC 0.846; 95% CI: 0.677–1.000; p = 0.021) with 100% sensitivity at the optimal cut-off, whereas CTSI scores demonstrated poor predictive ability (AUC 0.528; p = 0.852).

**Conclusion:**

BISAP demonstrated better predictive performance than CTSI in this cohort and may be a useful bedside tool for early risk stratification. CTSI remains valuable for assessing local pancreatic complications but has limited prognostic utility when used alone.

## INTRODUCTION

Acute pancreatitis (AP) is an inflammation of the pancreas, an acute gastrointestinal emergency, which affects multiple organ systems.^1^ The clinical presentation is heterogeneous; it can present as mild or progress to severe necrotizing pancreatitis associated with persistent organ dysfunction. Although most patients present with mild disease, almost 20% can turn into severe pancreatitis with consequential complications, prolonged hospitalization, and a notable risk of death.^2^ Given this, early identification of patients with high risk of progression to Severe Acute Pancreatitis (SAP) is critical for acute clinical decision making, and risk stratification in the deployed critical care approach to monitoring and treatment.^3^

There are multiple clinical, laboratory, and imaging-based scoring systems developed to assist in early risk stratification. Traditional systems like Ranson’s criteria and APACHE-II have their shortcomings, particularly when it comes to the complexity and need for repeated frequency of their assessments.^4^ However, the Bedside Index for Severity in Acute Pancreatitis (BISAP) score was designed to be a quick and simple-to-use algorithm that required five clinical parameters that can be made in the first 24 hours after a patient is hospitalized.^5^,^6^ After this score was designed and presented in the literature, it was demonstrated in a number of studies as a good predictor of complications and mortality in AP, and this predictive ability is especially helpful in resource-constrained settings.

Imaging is a key complementary system and the Computed Tomography Severity Index (CTSI) Scores are widely applied to assess pancreatic inflammation and necrosis.^7^,^8^ However, there are some limitations when it comes to the complete reliance on contrast-enhanced computed tomography (CECT), particularly with availability, exposure to radiation, risk of contrast, and, of course, the fact that there may be a lag in morphologic changes despite a deterioration in clinical status.^9^ There is also a lack of local data in comparison to the global literature on the BISAP and CTSI scores comparison. As both tools are measuring different facets of the disease, i.e. one is measuring the systemic derangement (BISAP scores) while the other is measuring the local derangement (CTSI scores), it is important to understand the relationship of these scoring systems to assist improved and cost effective clinical decision making.^10^ The aim of this study was to compare the predictive ability of CTSI and BISAP scores for the outcome of severe disease in acute pancreatitis.

## METHODOLOGY

This retrospective cross-sectional study was conducted in the Department of Radiology, Lady Reading Hospital–MTI, Peshawar, using patients records from January 2024 to December 2024. The data was extracted from the Hospital Management Information System (HMIS). A structured proforma was used to collect the demographic details, laboratory data, components of the BISAP scores, CT scan findings, CTSI score, complications, and outcomes. CT scans were performed on a Toshiba Aquilion Prime 160-slice scanner using intravenous contrast with 0.5 mm slice thickness.

### Ethical approval:

The Institutional Review Board (IRB) at Lady Reading Hospital–MTI (Approval No.: 470/LRH/MTI, dated 05/09/25) has approved this study. Since this is a retrospective study using anonymized data, the IRB approved a waiver of the requirement for informed consent.

### Sample size and Sampling technique:

This was a retrospective record-based diagnostic accuracy study, all eligible patients with acute pancreatitis who met the inclusion criteria during the study period were included. A consecutive sampling approach was used, resulting in a total sample of 99 patients.

### Inclusion Criteria:

- Patients who had a diagnosis of acute pancreatitis, as defined by the Revised Atlanta Criteria (2012).
- Patients who had clinical and laboratory data sufficient to allow for BISAP scoring within 24 hours.
- Patients who underwent a CT abdomen scan for acute pancreatitis severity assessment. CT was performed as clinically indicated, typically after 48–72 hours of admission.

### Exclusion Criteria:

- Patients who had chronic pancreatitis.
- Patients with incomplete laboratory or imaging records.
- Patients with an alternative diagnosis on CT scan that mimics the signs and symptoms of acute pancreatitis.

### Outcome measures:

The primary outcome variables were the severity of the acute pancreatitis and in-hospital mortality. The CTSI and BISAP scores were calculated using Fig.1. The Revised Atlanta Classification of 2012 indicates that a person can have mild, moderately severe, or severe acute pancreatitis.

**Fig.1:**
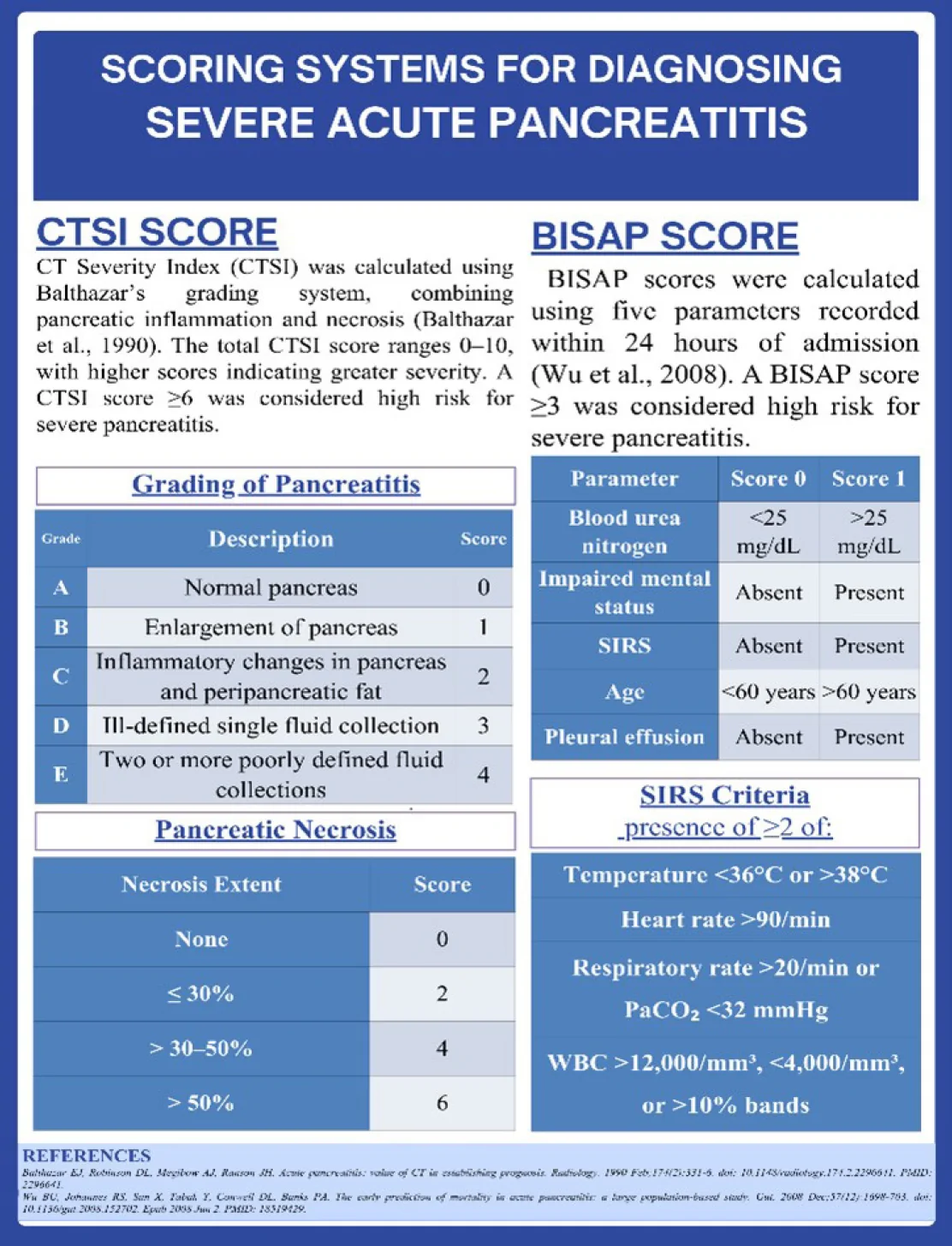
Parameters for assessment of severity in acute pancreatitis using BISAP and CT Severity Index (CTSI) scores.

### Statistical analysis:

Data were analyzed using SPSS version 25. Quantitative variables were expressed as mean ± standard deviation (SD), and categorical variables as frequencies and percentages. ROC curve analysis was used to evaluate the predictive accuracy of BISAP and CTSI scores for severe outcomes. A p-value <0.05 was considered statistically significant. From the ROC curve, we calculate the optimal cut-off values as those coordinates for which the corresponding Youden index (sensitivity + specificity - 1) is at a maximum.

## RESULTS

A total of 99 patients were included in the analysis, with a mean age of 42.5 ± 17.3 years. Most participants were female (Table-I), and the most common association found was gallstone disease (Table-I). According to BISAP scoring, 20.2% patients exhibited severe pancreatitis, whereas CT severity index classified 35.4% (Table-I) of patients as having severe acute pancreatitis, reflecting a higher proportion of radiological severity compared with bedside clinical scoring.

**Table-I:** Demographic & Clinical Characteristics of Study Population (n = 99).

Clinical and radiological characteristics	Category	Value
Gender	Male	33 (33.3%)
	Female	66 (66.7%)
Etiology	Gallstones	49 (49.5%)
	Hypertriglyceridemia	1 (1.0%)
	History of ERCP	8 (8.1%)
BISAP Scores Category	<3 (mild disease)	79 (79.8%)
	≥3 (severe disease)	20 (20.2%)
CTSI Scores Category	<6 (mild /moderate disease)	64 (64.6%)
	≥6 (severe disease)	35 (35.4%)

BISAP scores: bedside index of severity scores in acute pancreatitis; CTSI Scores: computed tomography severity index scores.

Based on the Computed Tomography Severity Index (CTSI), severe acute pancreatitis (radiological severity defined as CTSI 6–10) was identified in 35 patients (35.5%), as shown in Table-II. Among patients with radiologically severe disease (CTSI 6–10), the most frequent score was CTSI 6 (27.3%). Within this group, 40.5% of patients were classified as having severe acute pancreatitis (SAP) according to the revised Atlanta Classification. The overall mortality rate in the cohort was 4% (4/99). Notably, 50% of deaths occurred in patients with a CTSI score of six; however, this finding should be interpreted cautiously due to the small number of mortality events.

**Table-II:** Severity of Acute Pancreatitis and Mortality According to CTSI Scores (n = 99).

CTSI	No. of Patients n (%)	SAP n (%)	Mortality n (%)
2	33 (33.3%)	9 (24.3%)	1 (25.0%)
3	8 (8.1%)	3 (8.1%)	1 (25.0%)
4	22 (22.2%)	8 (21.6%)	0 (0.0%)
5	1 (1.0%)	1 (2.7%)	0 (0.0%)
6	27 (27.3%)	15 (40.5%)	2 (50.0%)
8	4 (4.0%)	1 (2.7%)	0 (0.0%)
10	4 (4.0%)	0 (0.0%)	0 (0.0%)

CTSI: computed tomography severity index; SAP: Severe acute pancreatitis according to revised atlanta classification.

In patients with lower CTSI scores (2–5), severe acute pancreatitis was less frequent but still observed, particularly in those with CTSI scores of two and four. Although CTSI scores of eight and 10 represent higher radiological severity, fewer cases of severe acute pancreatitis were observed in these categories. This finding is likely attributable to the small number of patients in these groups rather than a true reduction in clinical severity.

A progressive increase in disease severity was observed with rising BISAP scores (Table-III). Severe acute pancreatitis was most common with a BISAP score of 2 (37.8%), but was also observed in higher scores (Table-III). Mortality was confined to BISAP scores two and above but there were no deaths in scores zero and one. Hence, increasing BISAP scores is associated with higher disease severity and increased mortality in acute pancreatitis.

**Table-III:** Severity of Acute Pancreatitis and Mortality According to BISAP Scores (n = 99).

BISAP Scores	Number of patients n (%)	SAP n (%)	Mortality n (%)
Score-0	15 (15.2%)	2 (5.4%)	0 (0.0%)
Score-1	39 (39.4%)	11 (29.7%)	0 (0.0%)
Score-2	25 (25.3%)	14 (37.8%)	2 (50.0%)
Score-3	16 (16.2%)	8 (21.6%)	0 (0.0%)
Score-4	3 (3.0%)	2 (5.4%)	1 (25.0%)
Score-5	1 (1.0%)	0 (0.0%)	1 (25.0%)

BISAP Scores: bedside index of severity scores in acute pancreatitis; SAP: severe acute pancreatitis.

ROC analysis confirmed that the BISAP score had better discriminate predicting severe acute pancreatitis compared to the CTSI scores. BISAP scores had an AUC = 0.667 (95% CI: 0.559–0.775; p = 0.006), reflecting a statistically significant discrimination, while CTSI scores has AUC = 0.571 (95% CI: 0.456–0.686; p = 0.239) reflects a non-significant discrimination (Fig.2). Overall, these findings suggest that BISAP may provide better early risk stratification, while CTSI shows limited predictive performance in this cohort.

**Fig.2:**
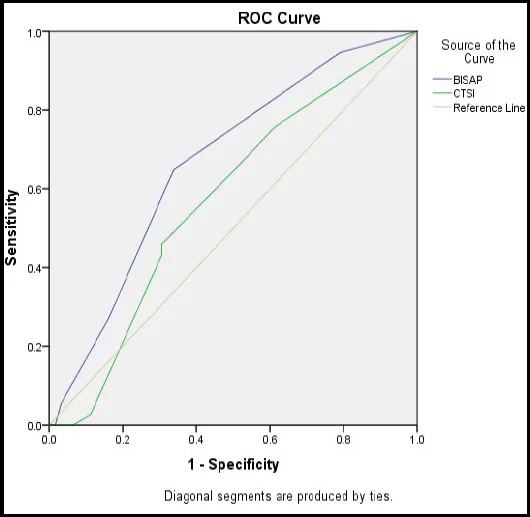
Comparison of BISAP and CTSI Scores in predicting severity.

ROC analysis for mortality prediction showed that the BISAP scores demonstrated better prognostic performance for this cohort as shown in Fig.3. BISAP scores achieved an AUC of 0.846 (95% CI: 0.677–1.000; p = 0.021), with 100% sensitivity and 56.8% specificity at the optimal cut-off (≥1.5), resulting in an NPV of 100%. In contrast, CTSI scores showed poor discriminatory ability, with an AUC of 0.528 (95% CI: 0.248–0.807; p = 0.852) and only 50% sensitivity and 41.1% specificity at the cut-off ≥3.5 as shown in Table-V. These findings indicate that BISAP showed better discriminatory performance for mortality prediction in acute pancreatitis than CTSI.

**Fig.3:**
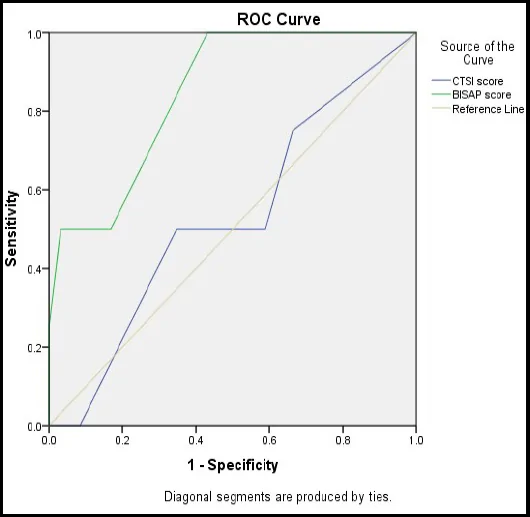
Comparison of BISAP and CTSI scores in predicting mortality.

**Table-IV:** Diagnostic Accuracy of CTSI and BISAP Scores for Predicting Severe Acute Pancreatitis (n = 99).

Scores (Cut-off)	Sensitivity	Specificity	PPV	NPV	AUC	95% CI	p-value
CTSI ≥ 3.5	67.6	46.8	43.1	70.7	0.571	0.456 – 0.686	0.238
BISAP ≥ 1.5	64.9	66.1	39.0	86.2	0.667	0.559 – 0.775	0.006

**Table-V:** Diagnostic Accuracy of CTSI and BISAP Scores for Predicting Mortality in Acute Pancreatitis (n = 99).

Scores (Cut-off)	Sensitivity	Specificity	PPV	NPV	AUC	95% CI	p-value
CTSI ≥ 3.5	50%	41.1%	21.4%	70.5%	0.528	0.248 – 0.807	0.852
BISAP ≥ 1.5	100%	56.8%	42.4%	100%	0.846	0.677 – 1.000	0.021

## DISCUSSION

In the present study, BISAP demonstrated superior predictive performance compared with CTSI for severe acute pancreatitis and in-hospital mortality. In our cohort, BISAP had a better ability to distinguish between severity and mortality outcomes on ROC analysis, indicating that early bedside clinical assessment may have a better prognostic value than radiological severity scoring alone. Since BISAP focuses primarily on capturing early systemic physiological disturbances, whereas CTSI focuses primarily on evaluating local morphological pancreatic changes, this distinction probably reflects the distinct constructs assessed by the two tools.

Our findings are consistent with previous studies. According to Jingoniya et al., BISAP outperformed CT-based assessment in terms of its early prognostic value and its usefulness as a disease severity predictor.^11^ In a similar context, Khattak et al. discovered that BISAP had high diagnostic accuracy in identifying severe acute pancreatitis in a Pakistani population using the Revised Atlanta Classification, demonstrating its suitability for use in local clinical settings.^12^,^13^ Chen et al. also demonstrated that BISAP is a reliable predictor of severity and adverse prognosis in acute pancreatitis, particularly because of its simplicity and early bedside applicability.^14^ All of these results show that BISAP is a useful clinical tool for early risk stratification.

There are a number of possible explanations for the relatively lower predictive performance of CTSI in our study. CTSI is dependent on radiological findings, particularly the presence and extent of pancreatic necrosis, which may not be fully apparent in early imaging. The significance of standard imaging intervals was emphasized by Huang et al., who pointed out that the timing of the CT acquisition had a significant impact on the predictive value of CTSI.^15^-^17^ In addition, the fact that CTSI primarily reflects local anatomical disease burden rather than systemic organ dysfunction and the interobserver variability in CT interpretation may make it less effective for early prognostication.^18^-^20^

In our cohort, BISAP performed better than CTSI when it came to mortality prediction. However, due to the fact that only four mortality events were recorded, statistical precision was limited so this finding should be interpreted with caution. Similar findings from previous studies indicate that bedside clinical scoring systems can be used to predict mortality.^21^ Cazacu et al. found BISAP to be comparable to other prognostic tools for mortality assessment in acute pancreatitis.^22^ Lavekar et al. also emphasized the utility of clinical scoring systems for mortality risk prediction in acute pancreatitis management.^23^ Although larger studies are required for a more robust mortality analysis, these observations support the clinical relevance of early bedside assessment. The different pathophysiologies that the BISAP and CTSI assess may also reflect the disparity in performance. Instead of just local pancreatic morphological changes, severe outcomes in acute pancreatitis are frequently caused by early systemic inflammatory response syndrome, persistent organ dysfunction, and metabolic abnormalities.^24^,^25^ As a result, a bedside score that takes into account both laboratory and clinical data may be more effective at identifying early disease progression.

### Strengths:

The application of the Revised Atlanta Classification for standardized severity assessment, the comparison of both clinical and radiological prognostic tools within the same patient cohort, and the use of real-world tertiary care hospital data.

### Limitations:

The retrospective, single-center design might make it harder to generalize. CTSI performance may have been affected by the inconsistent timing of CT imaging. Additionally, the strength of predictions regarding mortality is limited by the small number of deaths.

## CONCLUSION

In this cohort, BISAP outperformed CTSI in predicting severe acute pancreatitis and in-hospital mortality, indicating its usefulness as a bedside tool for early risk stratification. CTSI may supplement clinical evaluation in patient management and remains useful for assessing local pancreatic complications. However, due to the retrospective design of the study, the limited number of mortality events, and the variability in CT imaging timing, these findings should be interpreted with caution. In acute pancreatitis, an integrated approach that incorporates clinical and radiological evaluation may provide a more comprehensive evaluation.
